# Long-term cognitive outcome of Alzheimer’s disease and dementia with Lewy bodies: dual disease is worse

**DOI:** 10.1186/s13195-017-0272-8

**Published:** 2017-06-27

**Authors:** Frédéric Blanc, Rachid Mahmoudi, Thérèse Jonveaux, Jean Galmiche, Gilles Chopard, Benjamin Cretin, Catherine Demuynck, Catherine Martin-Hunyadi, Nathalie Philippi, François Sellal, Jean-Marc Michel, Gregory Tio, Melanie Stackfleth, Pierre Vandel, Eloi Magnin, Jean-Luc Novella, Georges Kaltenbach, Athanase Benetos, Erik A. Sauleau

**Affiliations:** 1 Memory Resource and Research Centre (CM2R), Geriatrics Day Hospital, Geriatrics Department, University Hospital of Strasbourg, 21 rue David Richard, 67091 Strasbourg Cedex, France; 2 University of Strasbourg and French National Centre for Scientific Research (CNRS), ICube Laboratory and Fédération de Médecine Translationnelle de Strasbourg (FMTS), Team Imagerie Multimodale Intégrative en Santé (IMIS)/Neurocrypto, Strasbourg, France; 3 University of Strasbourg, Laboratory of Biostatistics and French National Centre for Scientific Research (CNRS), ICube Laboratory, Team Modèles, Images et Vision (MIV), Strasbourg, France; 4 Geriatrics Department, Centre Hospitalier Universitaire Reims, Memory Resource and Research Centre (CM2R) Champagne-Ardenne, Reims, France; 5 Geriatrics Department, Centre Hospitalier Universitaire Nancy, Université de Lorraine, Memory Resource and Research Centre (CM2R) Lorraine, Nancy, France; 6 Psychiatry Department, Centre Hospitalier Universitaire Besançon, Memory Resource and Research Centre (CM2R) Franche Comté, Besançon, France; 7 Neurology Department, Centre Hospitalier Universitaire Besançon, Memory Resource and Research Centre (CM2R) Franche Comté, Besançon, France; 8 Association pour le Développement de la Neuropsychologie Appliquée (ADNA), Besançon, France; 9 Geriatrics Department and Neurology Department, Centre Hospitalier Général (CHG) de Colmar, Memory Resource and Research Centre (CM2R) Alsace, Colmar, France; 10 Neurology Department, |Centre Hospitalier Général (CHG) de Colmar, Memory Resource and Research Centre (CM2R) Alsace, Colmar, France

**Keywords:** Dementia with Lewy bodies, Alzheimer’s disease, Alzheimer’s dementia, Lewy body disease, MMSE, Outcome

## Abstract

**Background:**

Longitudinal studies of dementia with Lewy bodies (DLB) are rare. Clinically, DLB is usually considered to worsen into Alzheimer’s disease (AD). The aim of our study was to compare the rate of the cognitive decline in DLB, AD, and the association of the two diseases (AD + DLB).

**Methods:**

Using the Regional Network for Diagnostic Aid and Management of Patients with Cognitive Impairment database, which includes all the patients seen at all memory clinics (medical consultation and day hospitals) in four French regions, and beta regression, we compared the longitudinal the Mini-Mental State Examination scores of 1159 patients with AD (*n* = 1000), DLB (*n* = 131) and AD + DLB (association of the two) (*n* = 28) during follow-up of at least 4 years.

**Results:**

The mean follow-up of the patients was 5.88 years. Using beta regression without propensity scores, the comparison of the decline of patients with AD and patients with DLB did not show a significant difference, but the decline of patients with AD + DLB was worse than that of either patients with DLB (*P* = 0.006) or patients with AD (*P* < 0.001). Using beta regression weighted by a propensity score, comparison of patients with AD and patients with DLB showed a faster decline for patients with DLB (*P* < 0.001). The comparison of the decline of patients with AD + DLB with that of patients with DLB (*P* < 0.001) and patients with AD (*P* < 0.001) showed that the decline was clearly worse in the patients with dual disease.

**Conclusions:**

Whatever the analysis, the rate of decline is faster in patients with AD + DLB dual disease. The identification of such patients is important to enable clinicians to optimise treatment and care and to better inform and help patients and caregivers.

## Background

Alzheimer’s disease (AD) and dementia with Lewy bodies (DLB) are the two main neurodegenerative diseases responsible for dementia and account, respectively, for 70–80% and 15–20% of neuropathologically defined cases [1]. Diagnostic classification of DLB is based on revised consensus criteria, with the core features being: (1) recurrent visual hallucinations, (2) cognitive fluctuations and (3) spontaneous motor features of parkinsonism [1]. The presence of two or three of these core signs is sufficient for a diagnosis of probable DLB [1] at the stage of dementia. The outcome of patients with DLB is known to impact survival more than AD [2], particularly when patients have autonomic dysfunction [3].

The cognitive outcome of AD and DLB has previously been measured in longitudinal studies with fewer than 200 patients, with contradictory results. Some demonstrated a faster rate of decline for DLB than for AD [4, 5]. One study demonstrated a faster rate of decline solely for patients with dual disease (AD + DLB) compared with patients with either AD or DLB [6, 7]. However, most studies showed a similar rate of decline in patients with AD and patients with DLB. Thus, a recent meta-analysis of six studies in which researchers used the Mini Mental State Examination (MMSE), the rate of decline showed no significant difference between patients with AD and patients with DLB (annual declines of 3.4 and 3.3 MMSE points, respectively) [8]. The biggest study, with 315 patients (AD, *n* = 252; DLB, *n* = 63), likewise showed no difference in terms of cognitive outcome between AD and DLB [9].

To the best of our knowledge, no study has previously been done using data of a naturalistic longitudinal cohort. The primary aim of this study was thus to compare patients with AD, patients with DLB, and patients with AD + DLB in terms of cognitive rate of decline using the MMSE score as the outcome measure in a naturalistic cohort from a group of regions in north-eastern France.

## Methods

### Study design

Patients were consecutively recruited via the database of the Regional Network for Diagnostic Aid and Management of Patients with Cognitive Impairment (RAPID-Fr network), where all memory clinics in the French regions of Alsace, Champagne-Ardenne, Lorraine (starting from 2016, these three regions are known as “Région Grand Est”) and Franche-Comté register all patients who consult for cognitive complaints [10]. According to the French National Institute for Statistics and Economic Studies, at 1 January 2012, these four regions had a population of 8,307,000, corresponding to 12.6% of the population of France. Between 2003 and 2016, 222,202 consultations (by geriatricians, neurologists and psychiatrists) or geriatrics day hospital visits were recorded in the RAPID-Fr database for 100,698 patients. All memory clinics (including neurologists in liberal, memory centres and tertiary memory centres named *memory resource and research centres* [CM2R]) in the four French regions participate in the RAPID-Fr database, and all have been validated by the French Ministry of Health. We extracted from the database data recorded between 1 January 2003 and 1 July 2016 for patients with follow-up of at least 4 years and a declared diagnosis of AD alone, DLB alone or AD and DLB together (AD + DLB).

### Patients, assessments and diagnosis

**Fig. 1:**
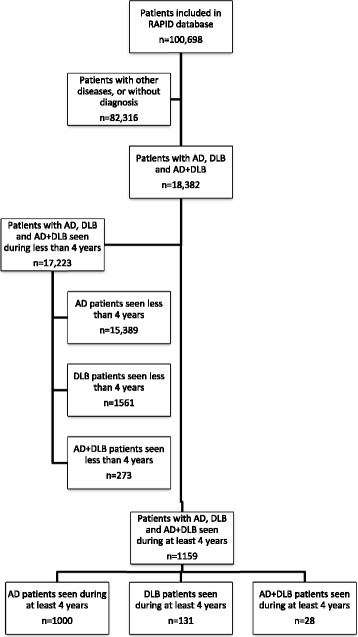
Flowchart of the present study on cognitive outcome in dementia with Lewy, Alzheimer’s disease and double disease. *AD* Alzheimer’s disease, *DLB* Dementia with Lewy bodies, AD + DLB Dual disease, *RAPID* Regional Network for Diagnostic Aid and Management of Patients with Cognitive Impairment

Among 4422 patients followed for at least 4 years for cognitive complaints (*see* Fig. 1), we found 1159 patients with AD (*n* = 1000 among 16,389 patients with AD seen at least once), DLB (*n* = 131 among 1692 patients with DLB seen at least once) and AD + DLB (dual disease) (*n* = 28 among 301 patients with AD + DLB seen at least once).

An aetiological diagnosis of the neurocognitive disorder for each patient was made using McKhann’s criteria for AD [11], McKeith’s criteria for DLB [12] and both sets of criteria for AD + DLB. The diagnosis was made by a multidisciplinary team at each memory centre, including geriatricians, neurologists, psychiatrists and neuropsychologists.

The MMSE version used was the French consensual version of the French Working Group on Cognitive Evaluation (GRECO) [13]. Among the sociodemographic data, we considered age in years, sex, and education level with five levels (no schooling, primary school level [equivalent to 1–5 years of education], *collège* [equivalent to secondary school level with 6–9 years of education], *lycée* [equivalent to secondary school level with 10–12 years of education] or university level [over 12 years of education]).

### Statistical analysis

Differences in demographic and clinical data at baseline were assessed for continuous variables using parametric analysis of variance. In post hoc analyses between diseases, we employed the Holm adjustment on *P* values. For categorical measures, χ^2^ tests were applied. To assess the difference between centres, Fisher’s exact test was used. For each test statistic, a probability value less than 0.05 was regarded as significant. Descriptive results are shown as mean ± SD for continuous variables and as number and percent for categorical variables.

Because MMSE has discrete values bounded by 0 and 30, we relied on beta regression for modelling a transformation of the score on (0, 1). MMSE was divided by 30 and, to exclude 0 and 1 values, transformed using the Smithson and Verkuilen method [14]. The transformed scores were assumed to be beta-distributed. The precision of these distributions was not modelled, but in the logit of its mean, we added several terms: a specific intercept for each disease (DLB, AD, and AD + DLB) and a specific temporal linear (on the logit scale) trend for each disease. All post hoc comparisons between intercepts and slopes were simultaneously inferred using contrasts and accurate corrections for type I error. On a logit scale, the values of the parameters are meaningless. When necessary, the back-transformation on the natural scale was achieved using the “expit” function (inverse function of logit) for the mean estimates and the delta method for the variance. We retrieved only the *P* value of different statistical comparisons (intercepts and trends between diseases), but, when relevant, we back-transformed means, SEMs and 5% CIs of parameters.

To deal with potential differences between subjects in each group at their time of inclusion in the study, we carried out two different analyses: (1) a “ground reality” beta regression as described above and (2) the same beta regression but using propensity scores based on demographic and clinical variables as weights (age, sex, education level). Calculation of this score was done using boosted logistic regression [15]. All statistical analyses were performed using the R version 3.2.3 statistical software package [16] with ad hoc packages (betareg, multcomp and twang). Results are shown to four significant digits.

## Results

### Subject characteristics and propensity scores

The demographic data for patients are summarised in Table 1. Subject groups differed among the three disease groups with regard to age, sex and education level.

**Table 1:** Baseline characteristics of patients in the three disease groups: dementia with Lewy bodies, Alzheimer’s disease and dual pathology

	DLB	AD	AD + DLB	*P* value
Age, years	74.4 ± 8.4	77.3 ± 8.1	79.1 ± 7.46	<0.001
Sex, male	52.2	34.9	43.6	<0.001
Education level^a^	3.1 ± 1.1	2.8 ± 1.1	2.9 ± 1.1	<0.001

Continuous variables are shown as mean (SD) and categorical variables as percent

*AD* Alzheimer’s disease, *DLB* Dementia with Lewy bodies

^a^ Considered as a discrete variable

Whereas ages were different between each of the three disease groups, sex and education level were different only between the AD and DLB groups. The propensity score was built on these three variables. It ranged between 1 and 55.26, with a median at 1.136, the 75th percentile at 1.286 and the 90th percentile at 4.894.

### MMSE outcome

Showing data retrieved from the beta regression weighted by propensity score (though results with unweighted regression were very similar), Table 2 summarises estimates for intercept of each disease on the natural scale (the value of MMSE at inclusion in the study). The intercept AD + DLB was intermediate (mean 20.81) between AD (19.34) and DLB (21.85). The intercept for AD was significantly lower than the intercept for AD + DLB (*P* < 0.001), which was significantly lower than that for DLB (*P* < 0.001).

**Table 2:** Estimation of value of Mini Mental State Examination at inclusion (intercept) for each disease on the natural scale (expressed as mean, SEM and 95% CI)

	Mean	SEM	95% CI	
AD	19.44	0.1522	19.15	19.79
DLB	21.67	0.1526	21.87	23.38
AD + DLB	20.41	0.1963	19.32	22.68

*AD* Alzheimer’s disease, *DLB* Dementia with Lewy bodies

**Fig. 2:**
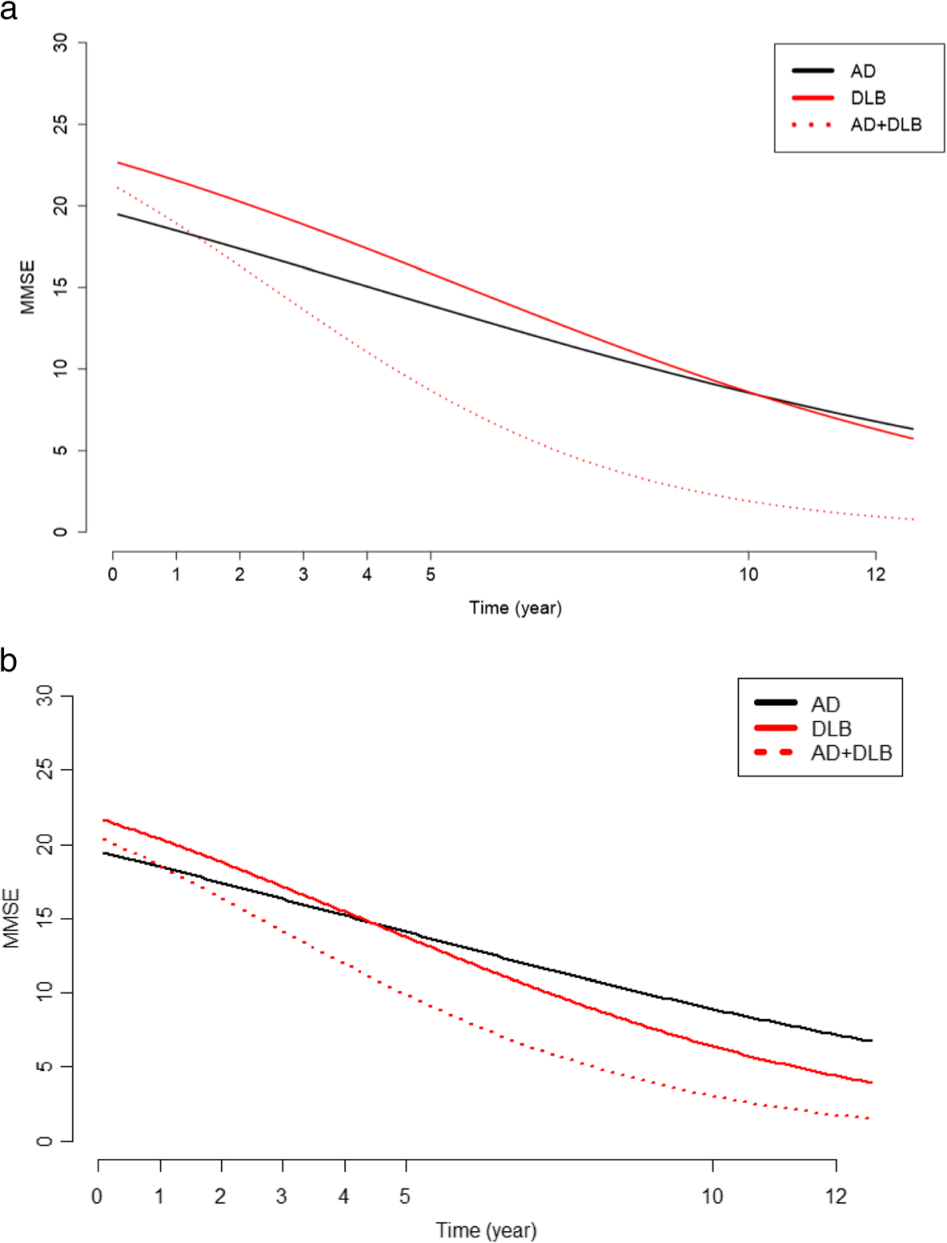
Estimated MMSE temporal evolution with beta regression without propensity score (**a**) and with propensity score (**b**) of patients with AD, patients with DLB and patients with AD + DLB. *AD* Alzheimer’s disease, *DLB* Dementia with Lewy bodies, *MMSE* Mini Mental State Examination

**Fig. 3:**
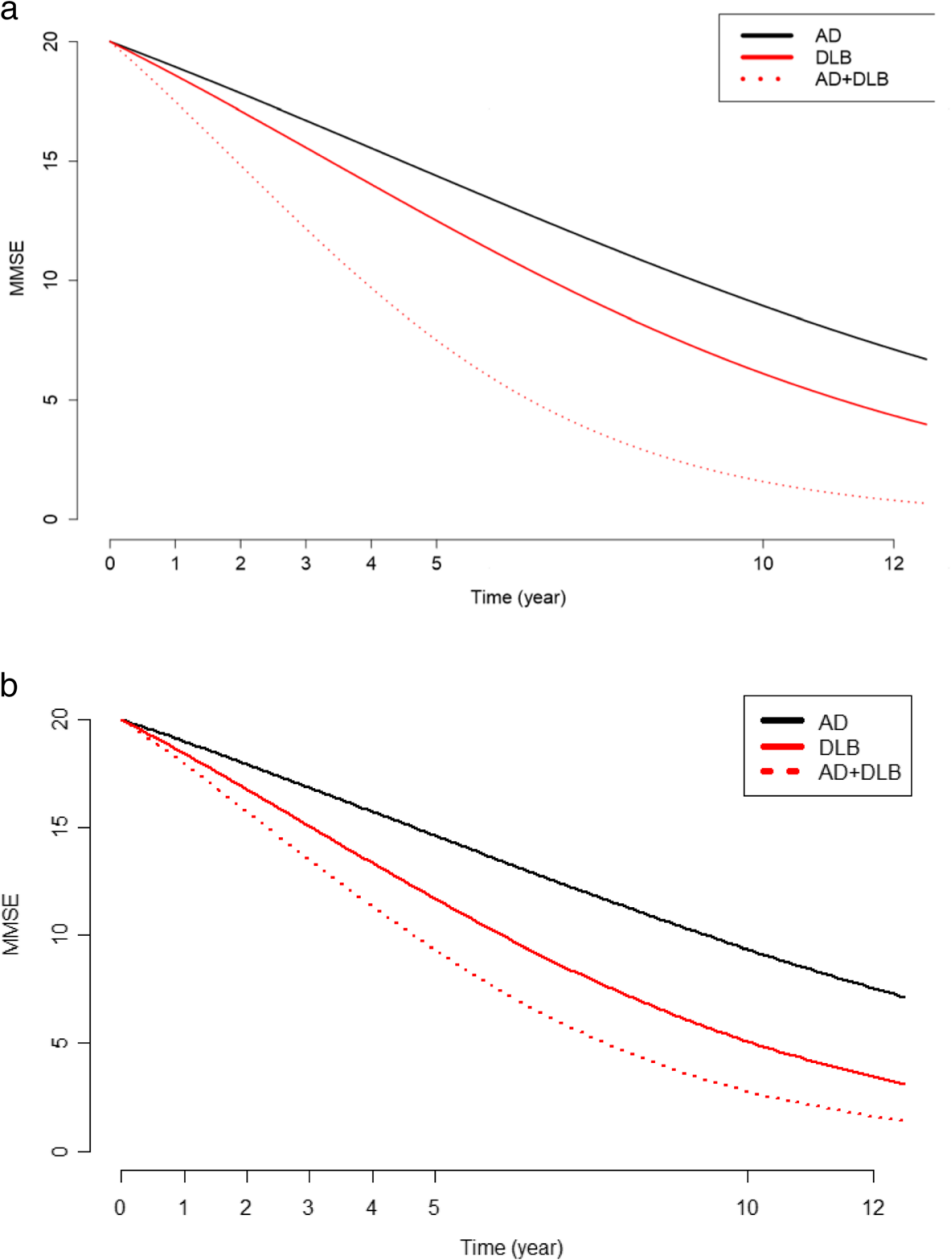
Estimated MMSE temporal evolution with beta regression without propensity score (**a**) and with propensity score (**b**) of patients with AD, patients with DLB and patients with AD + DLB, and assuming an initial MMSE value of 20. *AD* Alzheimer’s disease, *DLB* Dementia with Lewy bodies, *MMSE* Mini Mental State Examination

On the logit scale, all the three temporal trends (hence taking into account baseline differences in MMSE) were significantly decreasing (*P* < 0.001). When using beta regression without propensity score, we found that the results were as follows: −0.013 for AD, −0.017 for DLB and −0.030 for AD + DLB. These trends were not different between AD and DLB (*P* = 0.086), but the trend for AD + DLB was significantly lower with respect to the trend for AD (*P* < 0.001) and DLB (*P* = 0.006). When using beta regression with propensity score, we found that the results were as follows: −0.012 for AD, −0.019 for DLB and −0.025 for AD + DLB. All these trends were significantly different from each other (*P* < 0.001). Figure 2a (without propensity score) and Fig. 2b (with propensity score) show the three trends on a natural scale. Values of MMSE were difficult to compare on this scale. If we assumed that the MMSE values were 20 at inclusion using the beta regression without propensity score, for a subject with AD, the values were 18.94, 16.70, 14.39 and 8.94 at 1, 3, 5 and 10 years, respectively. For a subject with DLB with the same initial value and at the same times, the corresponding values were 18.59, 15.57, 12.51 and 6.11, and for a subject with AD + DLB, the corresponding values were 17.49, 12.17, 7.50 and 1.58. If we assumed that the MMSE values were 20 at inclusion using the beta regression with propensity score, for a subject with AD, the values were 18.99, 16.85, 14.63 and 9.36 at 1, 3, 5 and 10 years, respectively. For a subject with DLB with the same initial value and at the same times, the corresponding values were 18.43, 15.07, 11.70 and 5.09, and for a subject with AD + DLB, the corresponding values were 17.93, 13.50, 9.32 and 2.76 (*see* Fig. 3).When we considered a linear decrease of MMSE (which did not appear to be the case) and considered the rate of cognitive decline, we found that (1) in beta regression without propensity score, the MMSE decreased by 65.56% in 12 years (or 1.06 points per year) for AD, by 72.58% (or 1.37 points per year) for DLB and by 95.57% (or 1.68 points per year) for AD + DLB; and (2) in beta regression with propensity score, the MMSE decreased by 63.47% in 12 years (or 1.02 points per year) for AD, by 80.04% (or 1.45 points per year) for DLB and by 91.74% (1.56 per year) for AD + DLB. At the end of follow-up (a mean of 59.06 months for AD, 55.53 months for DLB and 50.69 months for AD + DLB), the mean MMSE values were 18.23 for DLB, 14.53 for AD and 14.04 for AD + DLB.

### Differences between centres for the rate of diagnosis

CM2Rs diagnosed proportionally more patients with DLB than memory centres or neurologists in liberal than patients with AD (Table 3). For the AD + DLB diagnosis, there was no statistically significant difference between the different types of centres when compared with AD diagnosis or DLB diagnosis. For details on AD + DLB diagnosis, *see* Table 4, which shows the clinical and paraclinical characteristics of the 19 patients diagnosed in the CM2R of Strasbourg.

**Table 3:** Centre responsible for diagnosis of each of the diseases (Alzheimer’s disease, dementia with Lewy bodies or both together)

	Alzheimer’s disease		Dementia with Lewy bodies		Alzheimer’s disease and dementia with Lewy bodies	
	*P* = 0.122^c^					
			*P* = 0.777^d^			
	*P* = 0.038^a^^,^^b^					
	*n*	%	*n*	%	*n*	%
Neurologist in liberal	30	3	3	2	0	0
Memory centre	300	30	26	20	4	14
Memory Resource and Research Centre	670	67	102	78	24	86
Total	1000	100	131	100	28	100

Fisher’s exact test

^a^Means statiscally significant difference in terms of type of centre responsible for the diagnosis of AD versus DLB

^b^The difference between Alzheimer's disease and dementia with Lewy bodies

^c^(NS), it is the difference between Alzheimer's disease and (AD and DLB)

^d^(NS), it is the difference between Dementia with Lewy bodies and (AD and DLB)

**Table 4:** Characteristics of the patients with dual disease (Alzheimer’s disease and dementia with Lewy bodies) from the Memory Resource and Research Centre of Strasbourg

Patients	Initial MMSE score	Memory storage deficit	Language deficit	Executive deficit	Visuospatial and/or visuoperceptive and/or visuoconstructive deficit	Visual hallucinations	Fluctuations	Parkinsonism	RBD	Hippocampal atrophy on brain MRI	CSF	FDG-PET or perfusion SPECT	AD or DLB diagnosed first	Years between diagnoses	DAT, flutemetamol, EEG
1	18	Yes	Yes	Yes	Yes^a^	Yes^a^	Yes^a^	Yes^a^	No	Yes (left)	3	Hypometabolism frontal, parietal, insular	AD	4	
2	22	Yes	Yes	Yes	Yes	Yes^a^	Yes^a^	No	Yes	Yes	ND	Hypometabolism temporal, occipital	AD	3	
3	24	No	No	Yes	Yes	Yes	Yes	Yes	No	Yes	3	hypometabolism frontal, temporal, parietal	DLB	1	Slow wave EEG
4	26	Yes	No	Yes	Yes	Yes	No	Yes	No	Yes	1 (P-Tau)	Hypometabolism frontal, temporal, parietal	AD	6	
5	25	Yes	Yes	Yes	ND	Yes	Yes	Yes	No	Yes	1 (Aβ)	hypometabolism frontal, parietal, occipital	AD	5	
6	24	Yes	No	Yes	Yes	Yes^a^	Yes^a^	No	No	Yes (left)	2 (tau, p-tau)	hypometabolism temporal, parietal, occipital	AD	5	
7	16	Yes	Yes	Yes	Yes	Yes^a^	Yes	No	No	Yes (right)	3	Hypometabolism temporal, parietal	AD	2	
8	24	Yes	No	Yes	No	Yes^a^	Yes^a^	Yes^a^	Yes^a^	No	2 (tau, p-tau)	ND	AD	4	
9	21	Yes	Yes	Yes	Yes	Yes	Yes	Yes	Yes	Yes	2 (p-tau, Aβ)	Hypoperfusion frontal, temporal, occipital	DLB	3	Pathological DAT slow wave EEG
10	25	Yes	Yes	Yes	Yes	Yes	Yes	Yes	No	No	ND	ND	AD	1	Pathological DAT slow wave EEG
11	25	Yes	No	Yes	No	Yes^a^	Yes^a^	Yes^a^	No	Yes	ND	ND	AD	6	
12	26	Yes	Yes	Yes	Yes^a^	Yes^a^	Yes^a^	Yes^a^	No	No	3	ND	AD	4	Pathological PET with flutemetamol
13	28	Yes	No	Yes	Yes	Yes^a^	Yes^a^	Yes^a^	No	Yes	ND	ND	AD	3	Pathological DAT scan
14	24	Yes	No	No	No	Yes^a^	Yes^a^	No	No	Yes	ND	Hypoperfusion temporal, occipital	AD	5	
15	20	Yes	No	Yes	Yes	Yes^a^	Yes	Yes	No	Yes	ND	Hypoperfusion temporal, parietal, insular	DLB	2	Pathological DAT scan
16	22	Yes	No	No	No	Yes^a^	Yes^a^	Yes^a^	No	Yes	3	Hypoperfusion temporal	AD	7	
17	26	Yes	No	Yes	No	No	Yes^a^	Yes^a^	Yes	No	ND	ND	AD	1	Pathological PET with flutemetamol
18	24	Yes	No	Yes	No	Yes^a^	Yes^a^	No	Yes^a^	Yes (right)	0	ND	AD	3	
19	24	No	No	Yes	No	Yes^a^	Yes	Yes^a^	No	Yes	0	Hypoperfusion temporal, parietal, insular	DLB	4	Pathological PET with flutemetamol

*Abbreviations: A*β Amyloid-β, *AD* Alzheimer’s disease, *CSF* Cerebrospinal fluid, *DAT* Dopamine transporter scan, *DLB* Dementia with Lewy bodies, *EEG* Electroencephalogram, *FDG-PET* Fluorodeoxyglucose positron emission tomography, *MMSE* Mini Mental State Examination, *MRI* Magnetic resonance imaging, *ND* Not done *p-tau* Phosphorylated tau, *RBD* Rapid eye movement sleep behaviour disorder *SPECT* Single-photon emission computed tomography

^a^ Symptoms arising during follow-up and not at the beginning

## Discussion

We report a clearly more significant cognitive decline in dual-disease patients, associating AD and DLB, than observed in patients with either pure AD or pure DLB. These results confirm the logical notion that the outcome is worse for patients with two neurodegenerative diseases than for patients with one neurodegenerative disease. There was no statistical difference between the decline in patients with DLB and the decline in patients with AD when the beta regression was without propensity score; however, when the beta regression was with a propensity score that took into account sex, education level and age, the decline was more marked in patients with DLB than in patients with AD.

Assuming a linear temporal evolution in MMSE score, the rate of cognitive decline was 1.02–1.06 points per year for AD, 1.37–1.45 points for DLB and 1.56–1.68 points for AD + DLB. However, because patients with DLB had a better MMSE score at the beginning of the study, the final MMSE score of patients with DLB was better than AD^−^ and also AD^+^DLB^−^ patients at the end of follow-up: 18.23 for DLB, 14.53 for AD and 14.04 for AD + DLB after a mean 56, 59 and 51 months of follow-up, respectively.

Nelson et al. previously demonstrated that patients with AD + DLB have a worse cognitive decline than patients with either pure AD or pure DLB [6, 7]. Their study was autopsy-proven, which explains why only 9 patients were included in the pure DLB group compared with 107 in the AD group and 27 in the AD + DLB group.

In our study, the three groups were different in terms of age, sex and education level. However, these differences are consistent with previous publications on AD and DLB [8]. Thus, in our cohort, there were more women in the AD group than in the DLB group. The sex ratio in AD cohorts usually shows a predominance of women [17]. In contrast, there is either a predominance of men or a balanced sex ratio in DLB cohorts [18, 19]. In our cohort, the AD + DLB group was the oldest. There is an increase in the reported prevalence of clinical AD as well as in DLB with age [20]. In our cohort, patients with DLB had a higher education level. However, the relationship between education level and dementia is unclear [21]. Most studies report a positive effect of education on cognitive performance but a lack of association with the rate of cognitive decline [22].

Thus, it would be quite artificial to consider only the beta regression with propensity score in our study and to conclude that the rate of cognitive decline in DLB is greater than the rate of decline in AD. As described above, the characteristics of patients with DLB differed from those of patients with AD, with, for instance, more women in the AD group than in the DLB group; these intrinsic characteristics must be preserved. This explains why the beta regression without propensity score is most likely a better reflection of the ground reality, showing that AD and DLB had roughly the same rate of decline. Moreover, in this respect, our study is consistent with previous studies, in particular with a meta-analysis of six studies [8], as well as with the previous biggest study [9], showing no difference in terms of cognitive decline between DLB and AD.

The diagnosis of AD + DLB is not crisply defined. That is the reason why most of the patients diagnosed as AD + DLB came from CM2R (tertiary memory clinic; 86%), and particularly from Strasbourg (68%) (*see* Table 4), which specialises in patients with DLB. Interestingly, the diagnosis of one of the diseases was done before the other one; thus, AD was usually first diagnosed (79%), and then DLB was diagnosed in addition to AD. Therefore, to diagnose patients with AD + DLB, the clinician had to be demanding of himself: Systematic interrogation of fluctuations, visual hallucinations and RBD, as well as a search for parkinsonism, was done even if the patient was previously diagnosed with AD. In the same way, if the first diagnosis was DLB (21%), the search for AD had to be done, particularly if on neuropsychological tests a memory storage deficit was found. In this situation, hippocampal atrophy raised interest in arguing for an AD diagnosis, but cerebrospinal fluid (CSF) analysis was clearly of importance [23].

Our study has two advantages: It is the first study with more than 350 patients (*n* = 1159), and it is the first naturalistic study including patients of all the memory clinics within a coherent geographical area (four large regions in north-eastern France). Our study has some limitations. Firstly, we do not have any autopsy verification of the patients. Thus, we cannot exclude the possibility that an incorrect classification of patients may have confounded the results. However, we used McKeith’s criteria, which have excellent specificity (greater than 95%) [24, 25] when compared with the gold standard neuropathological diagnosis. Moreover, the use of an autopsy series may overestimate the rate of decline owing to survival bias, because slower-progressing cases are less likely to have come to autopsy [5]. Secondly, the data used in this study were not based on a harmonised clinical procedure. However, though the MMSE was normed in France according to a different procedure, all the memory clinics involved in this study used the MMSE consensual version which was established by GRECO [13]. Similarly, there are French guidelines for the diagnosis of AD and DLB which require the use of a neuropsychological assessment and brain magnetic resonance imaging, as well as CSF analysis, single-photon emission computed tomography and positron emission tomography in the case of a difficult diagnosis [26]. Thus, CSF analysis is usually used to diagnose AD and DLB in France, including in our regions [23, 27]. Thirdly, more than 90% of the patients with AD, DLB or AD + DLB seen in our memory clinics were followed less than 4 years. This is due to the fact that the RAPID-Fr database was progressively implemented in the different memory clinics starting in 2003. Thus, it is possible that the number of patients with AD + DLB is more frequent in our regions because neuropathological data have demonstrated frequent associations between the two diseases [28].

## Conclusions

Our data suggest that patients with dual disease (AD + DLB) have a higher rate of cognitive decline and are consistent with previous studies showing that AD and DLB have a similar rate of decline. The identification of dual-disease patients is of importance to enable clinicians to optimise treatment and care and to better inform and help patients and caregivers. The next steps would be, firstly, to better understand the role of symptomatic treatment such as cholinesterase inhibitors or memantine in the two diseases, secondly to explore the functional outcome of these patients, and thirdly to explore the cognitive outcome of patients at the prodromal stage of AD, DLB and AD + DLB.

## Abbreviations

AD: Alzheimer’s disease
Aβ: Amyloid-β
CM2R: Memory resource and research centre
CSF: Cerebrospinal fluid
DAT: Dopamine transporter scan
DLB: Dementia with Lewy bodies
EEG: Electroencephalogram
FDG-PET: Fluorodeoxyglucose positron emission tomography
GRECO: French Working Group on Cognitive Evaluation
MMSE: Mini Mental State Examination
MRI: Magnetic resonance imaging
p-tau: Phosphorylated tau
RAPID-Fr: Regional Network for Diagnostic Aid and Management of Patients with Cognitive Impairment
RBD: Rapid eye movement sleep behaviour disorder
